# Lessons learnt from comprehensive evaluation of community-based education in Uganda: a proposal for an ideal model community-based education for health professional training institutions

**DOI:** 10.1186/1472-6920-11-7

**Published:** 2011-03-01

**Authors:** Dan K Kaye, Wilson W Muhwezi, Ann N Kasozi, Steven Kijjambu, Scovia N Mbalinda, Isaac Okullo, Rose C Nabirye, Hussein Oria, Lynn Atuyambe, Sarah Groves, Gilbert Burnham, Andrew Mwanika

**Affiliations:** 1 Department of Obstetrics and Gynecology, School of Medicine, Makerere University College of Health Sciences, P.O. Box 7072, Kampala, Uganda; 2 Department of Psychiatry, School of Medicine, Makerere University College of Health Sciences, P.O. Box 7072, Kampala, Uganda; 3 Office of the Dean, School of Medicine, Makerere University College of Health Sciences, P.O. Box 7072, Kampala, Uganda; 4 Department of Nursing, School of Health Sciences, Makerere University College of Health Sciences, P.O. Box 7072, Kampala, Uganda; 5 Department of Dentistry, School of Health Sciences, Makerere University College of Health Sciences, P.O. Box 7072, Kampala, Uganda; 6 Department of Pharmacy, School of Health Sciences, Makerere University College of Health Sciences, P.O. Box 7072, Kampala, Uganda; 7 Department of Community Health and Behavioral Sciences, School of Public Health, Makerere University College of Health Sciences, P.O. Box 7072, Kampala, Uganda; 8 Department of Nursing, Johns Hopkins School of Nursing, Baltimore, Maryland, USA; 9 Department of International Health, Johns Hopkins Bloomberg School of Public Health, Baltimore, Maryland, USA

## Abstract

**Background:**

Community-based education (CBE) can provide contextual learning that addresses manpower scarcity by enabling trainees acquire requisite experiences, competence, confidence and values. In Uganda, many health professional training institutions conduct some form of community-based education (CBE). However, there is scanty information on the nature of the training: whether a curriculum exists (objectives, intended outcomes, content, implementation strategy), administration and constraints faced. The objective was to make a comprehensive assessment of CBE as implemented by Ugandan health professional training institutions to document the nature of CBE conducted and propose an ideal model with minimum requirements for health professional training institutions in Uganda.

**Methods:**

We employed several methods: documentary review of curricula of 22 institutions, so as to assess the nature, purpose, outcomes, and methods of instruction and assessment; s*ite visits *to these institutions and their CBE sites, to assess the learning environment (infrastructure and resources); in-depth interviews with key people involved in running CBE at the institutions and community, to evaluate CBE implementation, challenges experienced and perceived solutions.

**Results:**

CBE was perceived differently ranging from a subject, a course, a program or a project. Despite having similar curricula, institutions differ in the administration, implementation and assessment of CBE. Objectives of CBE, the curricula content and implementation strategies differ in similar institutions. On collaborative and social learning, most trainees do not reside in the community, though they work on group projects and write group reports. Lectures and skills demonstrations were the main instruction methods. Assessment involved mainly continuous assessment, oral or written reports and summative examination.

**Conclusion:**

This assessment identified deficiencies in the design and implementation of CBE at several health professional training institutions, with major flaws identified in curriculum content, supervision of trainees, inappropriate assessment, trainee welfare, and underutilization of opportunities for contextual and collaborative learning. Since CBE showed potential to benefit the trainees, community and institutions, we propose a model that delivers a minimum package of CBE and overcomes the wide variation in the concept, conduct and implementation of CBE.

## Background

Community-based education (CBE) has several definitions [1,2], but the core definition refers to learning that takes place in a setting external to the higher education institution. In the context of health professional education, CBE refers to instruction whereby trainees learn and acquire professional competencies in community settings. Such settings include general practices, communities, community health centres or rural hospitals [2] with the focus being learning about health services in the community, methods of health promotion, as well as social and economic aspects of illness [2]. Community-oriented programmes have several goals: to create more appropriate knowledge, skills and attitudes; to deepen trainees' understanding of health and illness; to enable trainees understand the health and social services; to promote interpersonal skills and multidisciplinary teamwork; and to deepen trainees' understanding of the contribution of social and environmental factors to causation and prevention of ill-health [2].

CBE goes beyond cognitive capacities and encompasses the social and emotional aspects of learning, increasing trainee' experience, confidence and competence [2-4] and improving awareness of community values and lifestyles of health workers in rural areas [5,6]. CBE increases trainees' interest in uptake of careers in rural practice [5]. Provision of support for community site tutors and faculty improves the quality of medical students' learning experiences during rural rotations [6]. Indeed, such exposure to the communities through community placements during CBE shapes trainees' values and perceptions of rural practice, eventually promoting ethics, professionalism and health professionals uptake of rural practice [2,7,8].

In Uganda, many health professional training institutions conduct some form of CBE. However, there is scanty information on the nature of the training: whether a curriculum exists, the objectives related to CBE, intended outcomes, curriculum content. There is also scanty information on the implementation of the CBE curriculum (training sites, instruction methods, activities conducted by trainees, roles played by training site supervisors and institution faculty); administration of CBE (financial, human and material resources involved); and challenges faced by the implementation of CBE. Most reports of community-based programmes have been conducted in developed countries, have been largely descriptive, and rarely have assessed outcomes such as learning and rural deployment [9-11]. Others have only analyzed a specific component of CBE: limited to trainees' experience of the training during community placement [12], students' evaluation of the teaching and learning environment [13], specific contexts such as medical emergencies [14], evaluation of CBE conducted by a single academic institution [15], or evaluation of the role of community preceptors [16]. No similar studies have been documented on CBE for health professionals in Uganda. Specifically, no studies have documented the effectiveness of CBE in ensuring contextual learning for health professional trainees. Though medical schools and other health professional training institutions adopted CBE as a strategy for medical education, and community placements as one of the training settings, there is very little published research that explores the actual training that is conducted, the resources that are needed to sustain training, or the learning activities undertaken by students in different environments in Uganda.

Our aim was to answer the following questions: What is the nature of CBE conducted? Does CBE promote learning? What challenges do institutions face in implementing CBE and what are potential solutions? The objective of the comprehensive assessment of CBE conducted in health professional training institutions in Uganda was two-fold: 1) To analyze the nature of CBE conducted by different institutions as well as challenges involved; 2) To analyze effectiveness of CBE in promoting learning and acquisition of competences for cadres at different levels (certificate, diploma, advanced diploma and degree). As a spin-off of this comprehensive evaluation, we wish to utilize lessons to propose an ideal model with minimum requirements for health professional training institutions in Uganda. Documentation of this information as well as potential solutions to identified challenges in running CBE programs might not only improve the training of health professionals but also assist in the long-term goal of improving the recruitment, deployment or retention of health workers to rural areas.

## Methods

Through the twinning grant with Johns Hopkins University, Makerere University conducted a comprehensive assessment of CBE implemented by 22 health professional training institutions in Uganda, from October 2009 through May 2010. This assessment involved documentary review of available curricula and other documents related to CBE at 22 health professional training institutions, so as to assess the nature, purpose, intended outcomes, instruction methods and methods of assessment. Site visits to these institutions and two of their CBE sites was also conducted to assess the available infrastructure and learning resources available at these sites.

In analyzing whether learning occurs during CBE, we assessed the following:

i) whether learning is competence-based or outcome-based (that is, students are able to demonstrate certain competences, and focusing or organizing the learning in the educational system around what is essential for all students to be able to demonstrate successfully at the end of their learning experiences, for instance, critical reflection, communication skills and clinical skills)

ii) whether learning during CBE is problem-based (whether real-life situations related to patient care are used to stimulate learning);

iii) whether learning during CBE is contextual (reality-based, outside-of-the-classroom experiences, which serve as a catalyst for students to utilize their disciplinary knowledge, are employed in real-life settings similar to where trainees will eventually work, for instance health centres or the community);

iv) whether CBE promotes collaborative learning (whether learning occurs in situations in which two or more students learn, or attempt to learn, something together, as pairs, small groups or whole class), and perform learning activities such as problem-solving using individual effort, joint effort or external effort;

v) whether reflection (critical appraisal of incidents and experiences) occurs;

vi) whether CBE promotes lifelong learning (stimulates trainees to keep up-to-date with current clinical management or professional care guidelines).

vii) Since students contribute to service during community placements, we assessed whether such service contributes to trainees' learning (that is, whether service improves critical reflection whereby critical incidents are noted, appraised, analyzed and used to develop individual or group learning plans).

viii) Lastly, in-depth and key-informant interviews were conducted with key people involved in running CBE at the institutions and CBE sites to assess how CBE was implemented, challenges experienced and perceived solutions. Interviews with alumni of these institutions and community members at the CBE sites offered insight into the impact of CBE, challenges in CBE implementation and how these challenges may be addressed. Content analysis [17] was applied to analysis of these interviews: familiarization; identifying a thematic framework; indexing; mapping and interpretation. The data was then clearly coded and the text was indexed by using descriptors alongside various passages in the transcriptions.

Ethics and research committees of Makerere University College of Health Sciences and Johns Hopkins School of Public Health approved the research. Approval to conduct the research was given by the administrators of all the health institutions that were assessed.

## Results

Table 1 shows the findings from review of the curriculum documents, the implementation of the curriculum, the instruction methods used and evaluation of collaborative learning. Some of the institutions had no curriculum or the curricula documents were deficient with regard to CBE. Even where CBE was indicated in the curricula, there was wide variation in its perception, conduct and implementation. Activities conducted, instruction methods used and learning environment (community sites used to provide opportunity for learning) varied greatly in similar institutions. Lectures, assignments and skills demonstrations were the main methods of instruction.

**Table 1:** Assessment of CBE at the 22 institutions evaluated

Characteristic	Number (Percentage)
*Evaluation of the CBE curriculum document*	
There is a CBE curriculum	20 (90.9)
The curriculum has goals and objectives	18 (81.8)
The curriculum has clear intended outcomes on CBE	16 (72.7)
The curriculum has an evaluation plan	9 (40.9)

*Implementation of CBE and resources available*	
There are community training sites	18 (81.8)
CBE site tutors are used	18 (81.8)
Learning takes place in the right context	18 (81.8)
Learning is self directed	15 (68.2)
There is immediate feedback to trainees	18 (81.8)
Libraries are available	6 (27.2)

*Assessing for collaborative/social learning*	
Live in community or at a hostel within the community	8 (36.3)
Work on a group project	19 (86.4)
Work on individual project	11 (50.0)
Learn within and participate in multidisciplinary teams	11 (50.0)
Write a group report	19 (86.4)
Write individual reports	17 (77.3)
Are linked to traditional medical practitioners during training	10 (45.5)

*Instruction methods*	
Lectures	20 (90.9)
Seminars	11 (50.0)
Workshops	8 (36.3)
Small groups	18 (81.8)
Learning problems	11 (50.0)
Case studies	10 (45.5)
Assignments	20 (90.9)
Skills demonstration (such as demonstration of how to vaccinate children, how to perform clinical examination, or how to perform venepuncture)	22 (100.0)

*The learning context*	
Urban/peri urban areas	15 (68.2)
District headquarters	7 (31.8)
Schools	15 (68.2)
Health centers or district hospitals	22 (100.0)
In homes	17 (77.3)
With Non-Government Organizations	9 (40.9)

Table 2 shows the activities which students are involved in to foster learning. Self-directed learning and group discussions were the main activities which trainees used to enhance learning. The findings show that considering the variety of learning sites to which trainees are exposed, CBE had the potential to benefit trainees with experiential and contextual learning, while providing a service to the CBE sites as well as the community. However, less than half of the institutions had a research component as part of the activities students are involved in during CBE, though trainees had prior skills and resources for conducting research. This indicates a missed opportunity for CBE programs.

**Table 2:** Learning, research and assessment of learning

Characteristic	Number (Percentage)
*Strategies to promote understanding and learning*	
Structured group discussions	20 (90.9)
Peer feedback	16 (72.7)
Mentorship	15 (68.2)
Self-directed learning	21 (95.5)
Peer assessment	13 (59.1)
Portfolios	7 (31.8)
Community projects including community diagnosis	15 (68.2)

*Research during community-based training*	
Trainees prepared with prior training in research methodology	16 (72.7)
Trainees prepared with prior training in data analysis	17 (77.3)
Trainees prepared with prior training in report writing	18 (81.8)
Trainees have facilities for literature review (print media)	13 (59.1)
Trainees have facilities for literature review (electronic resources)	7 (31.8)
There is a research component	10 (45.5)
There is operations research	5 (22.7)
Site tutors are involved (participate)	7 (31.8)
There is community diagnosis	9 (40.9)
Research is assessed and marks are awarded	9 (40.9)
Research is conducted as a follow up on community diagnosis	7 (31.8)
CBE sites/community get feedback on findings	6 (27.2)

*Assessment methods used*	
Activities are observed and assessment given with feedback	21 (95.5)
Using log books	10 (45.5)
Using oral reports (debriefing)	19 (86.4)
Using peer assessment	17 (77.3)
Using written reports	21 (95.5)
Using progressive examinations at the institutions	17 (77.3)
Through a summative examination at the institutions	20 (90.9)
Through presentation to a panel of examiners	6 (27.2)

Table 3 shows the overall social environment in the community and health facilities. The evaluation shows that there is wide variation in available resources to support CBE, which were shared by different institutions despite their being inadequate. Most deficits were in accommodation facilities, welfare, sundries and supplies. Transport to and within the community sites was a problem. Our findings demonstrate the challenges that need to be addressed (especially financial, supervisory and administrative constraints) and the missed opportunities to be rectified if the CBE learning environment is to be enhanced.

**Table 3:** Facilities at sites to facilitate CBE activities

Checklist of necessary facilities/environment for a CBE site		Present n (%)
**Student welfare**		
	Transport	15 (68.1)
	Accommodation	11 (50.0)
	Washing facilities	14 (63.6)
	Toilet facilities	15 (68.1)
	Food provision	13 (59.1)
	Cooking facilities	14 (63.6)
	Recreation/Leisure	7 (31.8)
	Observing house rules	12 (54.5)
	Security	14 (63.6)
**Health facility**		
	Community health activities	16 (72.7)
	Involvement of health workers in training	16 (72.7)
	Facilities for PBL	3 (13.6)
**Community**		
	Support from the local leadership	15 (68.1)
	Involvement of traditional healers	10 (45.5)
	Involvement of partner organizations	7 (31.8)
	Involvement of community based organizations	13 (59.1)
	Community awareness of CBE	14 (63.6)
	Community acceptance	16 (72.7)
	Relevance of CBE activities	15 (68.1)
	Involvement of community in CBE	14 (63.6)
	General attitude f community towards CBE	15 (68.1)

Though most interviewees felt the concept of CBE was right, there were many challenges in its conduct, which hinder adequate preparation and implementation. Some of the beneficial aspects of CBE identified were early contact with the community, improved team work in the trainees who leave, work and learn in small groups, improved interpersonal relationships and improved communication skills. That the CBE was outcome-based is indicated by some curricula having clear objectives, learning outcomes, desired competencies and clear implementation strategy. Collaborative learning is indicated by the individual, joint and external collaborations by students to promote learning. CBE offered opportunities for improved clinical, leadership, self-directed learning and research skills. As an outcome of this comprehensive assessment, we identified a critical need to propose an ideal CBE curriculum whose content and implementation would provide a minimum package of CBE. Indeed, such an ideal model might make available human resources with competence to address Uganda's current and evolving priority health problems.

We found that though many health professional training institutions in Uganda conduct some form of CBE, the content of the curricula and the extent to which they are implemented is variable. For institutions without a written curriculum, we found no clear explanation why this was so from interviewing the faculty. The tutors, faculty and community members interviewed were in agreement that CBE has the potential to benefit trainees (through providing a real-life training environment where the graduates may eventually work, such in health centres, rural hospitals or the community); the community (whose members benefit from the services of the trainees during the community placements and outreach activities); the institutions (which get a better training context for their students); and the staff at the community training sites (who gain from update of knowledge and skills from interaction and collaboration with staff from the training institutions). CBE also offered opportunities to conduct research on conditions that affect the community (through community diagnosis). Different descriptions were given to CBE in the different institutions, consequently, CBE was administered the differently depending on how it was defined and the resources available. The key personnel ranged from individuals (acting as coordinators), a committee of persons, or an administrative office (usually under the dean, academic registrar or principal tutor). Factors considered before CBE site selection and preparation included proximity, available infrastructure, uniqueness of the site in terms of services offered (such as maternal and child health and mental health), receptivity of health facility staff and community leadership, ability of the potential site to cater for trainee's welfare, and availability of willing personnel at health facilities to supervise trainees. For the latter, 18 (70%) of the institutions had neither a formal criteria for selection of community supervisors nor formal training of these people.

## Discussion

Our findings evaluated the potential effectiveness of CBE in enhancing training of health professions. The methods of evaluation used enable evaluation of the ''curriculum on paper'' (what is written about the curriculum in documents and what the implementers understand about curriculum goals and objectives), the ''curriculum in action'' (how the curriculum is implemented) and the ''curriculum as experienced''(what students actually do, how they study and outcomes of the learning) [15]. Our findings therefore indicate that CBE provides contextual learning for most of the institutions (reality-based, outside-of-the-classroom experience, such as in the health centres, the community, schools or at the district health office as well as working with NGOs at community level). Contextual learning provides learning experiences in contexts in which trainees are interested and motivated and therefore achieve more [18-20]. The learning process of CBE includes the learner's ability to gain and utilize acquired knowledge as well as solve problems, while developing collaborative skills, innovation skills, communication skills, critical reflection, teamwork and interpersonal relationships [2,18,20].

In our assessment, there were wide variations in the curriculum content even for public institutions that offer the same academic award. It was also unclear to some institutions how the curriculum was developed, implemented or evaluated. Developing a generic CBE curriculum should follow a systematic approach that is capable of integrating content area with educational theory and methodology, and indicate evaluation of the impact of this process [21-24]. When completed, the CBE needs assessment should make a strong argument for the need for the curriculum, identifying previously developed or validated methods.

Some of the CBE curricula documents were unclear about the learning outcomes or desired competences, so it was unclear if the actualized curriculum included them. From the interviews, this was still unclear as some tutors were not conversant with the curriculum content or implementation. Regarding content and outcomes of the ideal CBE curriculum, the professional competences should focus on theoretical and applied knowledge associated with practice, emphasizing values, skills and critical appraisal. This is essential as training and practice/service often occur in situations characterized by complexity, uniqueness, uncertainty and conflicting values [23]. In contrast to the curriculum on paper, the curriculum in action is how the intended curriculum is implemented in practice [25], while the actualised curriculum refers to what students actually do, how they study, what they believe they should be doing, the learning that occurs and the outcome of their learning [25].

While pre-placement orientation is essential for success of CBE, it was unclear whether all institutions conducted it. Most of the CBE curricula emphasized knowledge and professional skills. Whereas specialized knowledge and skills are clearly essential for practice, self consciousness (reflection) and continual self critique (critical reflection) are crucial to acquisition of competences [23]. Reflective and critically reflective practice, communication skills and interpersonal relations are vital professional skills for effective and efficient professional practice [24].

Few of the institutions employed Problem-based learning (PBL) as an instruction method. Regarding implementation strategies, PBL is ideal for CBE as it effectively brings out the desired competences of the ideal CBE curriculum. Though the exact format of PBL varies considerably, two key features are consistent: emphasis on learner-focused exploration of case-based problems and use of case histories to help students identify learning issues that become the focus of individual or group problem-solving. During CBE, trainees should be exposed to professionally meaningful situations or problems that bear strong resemblance to the situations they will be confronted with in their future profession, which enables acquisition of knowledge, clinical skills and problem-solving skills. Development of critical reflection and critical thinking as professional skills during CBE requires adoption of PBL as the ideal model of instruction [22-24]. Indeed, during CBE, individual trainees interpret their varied experiences in their own way and understanding, basing on what happens to them and what they see, hear and read. [26]. CBE provides a holistic picture of health and health systems in the community.

The institutions evaluated conducted assessment of CBE using different and diverse methods, with variations in reliability. For CBE, there is a challenge of designing assessment such that it adequately reflects the desired competences, the instruction methods and the eventual professional role, while making it retain reliability and validity across different settings and assessors [27,28]. During CBE, students are taught in diverse contexts by diverse groups of tutors with varied competences and skills as educators. In the curricula, institutions should be clear about what and why they need to assess and who will do the assessment [2]. Assessment strategies need to be developed to enhance and support learning as well as reliably measure performance with evidence of learning having taken place [29,30]. While both formative and summative assessment are essential, assessment of CBE requires development of portfolios by the trainees [29]. Portfolios facilitate evaluation of integrated and complex abilities taking into account the learning context [29]. Indeed, portfolios personalize the assessment process, incorporating important educational values, while supporting the important principle that assessment drives learning. Other suitable methods include: peer evaluation, which may ably assess individual student effort, interaction with the community, leadership, knowledge or subject matter; supervisors' checklists; feedback from community leaders; and individual and group reports.

### Limitations

We acknowledge several limitations. Firstly, the institutions were purposively sampled in order to try to achieve representativeness of rural versus urban institutions, private versus public, the different cadres trained and different academic level of training. There is no guarantee that the institutions that were not sampled have similar constraints or challenges. However, all the government-supported institutions are supposed to follow and implement a similar curriculum. Secondly, the assessment was conducted regionally by four different teams. Since the teams piloted the instruments, there may have been slight variations (across the four teams) in the way interviews were conducted, checklists performed or documents reviewed. Thirdly, no direct observation of conduct of CBE was performed, yet this should be done in an ideal CBE assessment [31,32]. However, the methods (documentary review, checklists and interviews) employed are reliable [31], and have been used by others [15]. Lastly, the ideal curriculum would require validation of its objective and content [31]. This step was conducted at a stakeholder meeting of the Ministry of Health, Ministry of Local Government, all the training institutions and community representatives.

## Conclusion

This assessment identified deficiencies in the design and implementation of CBE at several health professional training institutions, with major flaws identified in curriculum content, supervision of trainees, inappropriate assessment, trainee welfare, and underutilization of opportunities for contextual and collaborative learning. Despite similar goals, there is wide variation in the concept, conduct and implementation of CBE conducted by different health institutions in Uganda, even in presence of similar curricula. CBE, if well implemented and assessed, has the potential to ensure contextual learning in medical education.

## Competing interests

All authors (except GB, LWC and SG who are from Johns Hopkins University) are faculty of Makerere University College of Health Sciences where community-based education is part of the curricula for undergraduate training of health professionals, and all community sites assessed are used by the university. GB, LWC and SG are team members of the project: *Partnership for building the capacity of Makerere University to improve priority health outcomes in Uganda*, but have no competing interests.

## Authors' contributions

All co-authors were involved in the design of the study on comprehensive assessment of community-based education. All except LWC and GB participated in the data collection and data analysis. DKK wrote the first draft of the manuscript. All co-authors contributed by reviewing the subsequent drafts of the manuscript and approved the final version

## Appendix

### Proposal for a model CBE curriculum for training health professionals

#### Steps in developing and implementing the ideal CBE curriculum

Step 1: Problem identification and general needs assessment: Identification and critical analysis of the educational need or problem that will be addressed by the curriculum. This step requires substantial research to analyze what is currently being done by practitioners (such as alumni of the training institutions) and educators (that is, the current approach) and ideally what should be done by practitioners and educators to address the health care problem (the ideal approach) and therefore the performance gap. The general needs assessment is usually stated as the knowledge, attitude, and performance deficits that the curriculum will address.

Step 2: Needs assessment of targeted learners

Step 3: Goals and objectives: Overall goals and aims for the curriculum are written. Specific measurable knowledge, skill/performance, attitude, and process objectives are written for the curriculum.

Step 4: Educational strategies: A plan to maximize the impact of the curriculum, including content and educational methods congruent with the objectives, is prepared.

Step 5: Implementation: A plan for implementation, including timelines and resources required, is created. A plan for faculty development should be made to assure consistent implementation

Step 6: Evaluation and feedback: Learner and program evaluation plans are created. A plan for dissemination of the curriculum is made.

#### Perceived Ideal objectives of CBE for health professionals

The primary aim should be:

1 Trainees to acquire an understanding of health and disease, positioning the prevention and management of disease in the context of the whole individual in his or her place in the family, community and society.

2 To develop health professionals with an attitude to learning that is based on curiosity and knowledge exploration rather than passive knowledge acquisition. This enables trainees become reflexive learners who can identify their learning needs, seek the desired information and eventually become life long learners

3 To enable trainees integrate the theory and practical aspects, as well as the basic science and clinical/professional aspects of their training. This necessitates introduction of components of problem-based learning in CBE, whereby learning is based on real-life contexts

4 To ensure early direct contact with patients/clients and critical appraisal/analysis of their problems, and thereby trainees attain problem-solving skills

#### The curriculum components

The curriculum should:

1. Provide an ideal balance of factual information and practical skills in addition to providing core knowledge, skills and attitudes

2. Develop general competence (e.g. critical thinking, problem-solving, communication, leadership, teamwork, management)

3. Ensure early clinical contact while strengthening inter-professional collaboration, thereby providing a balance between hospital/community; curative/preventive aspects.

4. Cover the wider aspects of health care (e.g. medico-legal issues, health economics, political aspect of health systems and medical audit)

5. Ensure that methods of learning/teaching support aims of the curriculum, and likewise, ensure that assessment methods employed support and rhyme with aims of the curriculum (are they reliable in assessing the competences?)

#### Competences of the trainee

The minimum competences of the trainees should include:

1. Knowledge and skills in caring for the community's health with emphasis on primary care, disease prevention and promotion of healthy lifestyle, and in involving patients, families and communities in healthcare decision-making

2. Understanding the factors that influence health and disease at household level, as well the roles and responsibilities of different players in the healthcare system

3. Innovation and problem solving skills necessary to provide cost-effective care using technology appropriately

4. Skills in collection, analysis and utilization information on health systems

5. Understanding the role of the physical, social and political environment in health

6. Life long learning (continuous acquisition of knowledge depending on critical reflection and self-criticism to identify learning needs)

7. Professionalism (learning values of how a professional is expected to perform and relate with the community and all other players in the healthcare system)

#### Curriculum content related to CBE

The curriculum content should include:

1. Broad education in both the clinical and basic sciences and integration of CBE into the science of profession

2. Clearly defined objectives and intended outcomes of CBE

3. Emphasis on methods and processes of acquisition of knowledge, professional skills, lifelong learning skills, values and attitudes (such as indicating examples of what activities trainees should be involved in)

4. Emphasis on utilization of educational sites beyond the training institution (such as schools, health centres, community health projects or working with non-government organizations)

5. Emphasis on adapting training to meet the challenges produced by ongoing changes in the organization, structure, financing and delivery of health care

#### Roles of tutors, supervisors and faculty

1. To identify suitable teaching resources and to conduct feedback and trainee appraisal

2. To design educational programs for trainees as well as identify suitable problems to deliver effective educational events for learning

3. To utilize appropriate teaching methods that employ large and small groups

4. To provide individual assistance depending on student needs

5. To assess the trainees used a variety of assessment methods that include portfolios

6. To evaluate the CBE program in context of the health professional training course

## Pre-publication history

The pre-publication history for this paper can be accessed here:

http://www.biomedcentral.com/1472-6920/11/7/prepub
